# Green Biomass-Based Protein for Sustainable Feed and Food Supply: An Overview of Current and Future Prospective

**DOI:** 10.3390/life13020307

**Published:** 2023-01-22

**Authors:** Éva Domokos-Szabolcsy, Seckin Reyhan Yavuz, Edgard Picoli, Miklós Gabor Fári, Zoltán Kovács, Csaba Tóth, László Kaszás, Tarek Alshaal, Nevien Elhawat

**Affiliations:** 1 Department of Applied Plant Biology, University of Debrecen, Böszörményi Str. 138, 4032 Debrecen, Hungary; 2 Department of Plant Biology, Federal University of Viçosa, Viçosa 36570-900, Brazil; 3 Department of Biological and Environmental Sciences, Faculty of Home Economic, Al-Azhar University, Tanta 31732, Egypt; 4 Soil and Water Science Department, Faculty of Agriculture, Kafrelsheikh University, Kafr El-Sheikh 33516, Egypt

**Keywords:** biomass crops, RuBisCo, green-based protein, phytochemicals, vitamins, agrowastes

## Abstract

It is necessary to develop and deploy novel protein production to allow the establishment of a sustainable supply for both humans and animals, given the ongoing expansion of protein demand to meet the future needs of the increased world population and high living standards. In addition to plant seeds, green biomass from dedicated crops or green agricultural waste is also available as an alternative source to fulfill the protein and nutrient needs of humans and animals. The development of extraction and precipitation methods (such as microwave coagulation) for chloroplast and cytoplasmic proteins, which constitute the bulk of leaf protein, will allow the production of leaf protein concentrates (LPC) and protein isolates (LPI). Obtained LPC serves as a sustainable alternative source of animal-based protein besides being an important source of many vital phytochemicals, including vitamins and substances with nutritional and pharmacological effects. Along with it, the production of LPC, directly or indirectly, supports sustainability and circular economy concepts. However, the quantity and quality of LPC largely depend on several factors, including plant species, extraction and precipitation techniques, harvest time, and growing season. This paper provides an overview of the history of green biomass-derived protein from the early green fodder mill concept by Károly Ereky to the state-of-art of green-based protein utilization. It highlights potential approaches for enhancing LPC production, including dedicated plant species, associated extraction methods, selection of optimal technologies, and best combination approaches for improving leaf protein isolation.

## 1. Introduction

Humans and animals consume protein mainly as a source of nitrogen and proteinogenic amino acids required for the synthesis of new biomolecules and as an energy source, to a lesser extent, providing four calories per gram of protein. The quality and nutritional value of protein depend heavily on its amino acid composition, particularly with regard to the nine indispensable amino acids (i.e., phenylalanine, valine, threonine, tryptophan, methionine, leucine, isoleucine, lysine, and histidine) [1]. 

Protein-energy malnutrition (kwashiorkor) is still the primary worldwide health problem. After World War II, the nutritional problems in developing countries received increased attention, and several low-income countries reported cases of severe kwashiorkor. Consuming protein comes first on the list of priorities. The discovery of novel protein sources, many of which came from unexpected assets, such as single-cell proteins, fish protein concentrates, and leaf protein concentrates, were prompted by this, as were efforts to add extra protein to meals [2].

The proteins from green leaves were first isolated in 1773 by the French biochemist H.M. Rouelle, although the proteins were not fully understood at the time [3]. He used a simple mortar and pestle to press the juice of the leaves. From the green juice, he obtained a green curd by heating it until his finger could no longer be held in the solution. Then the green curd was filtered off, and the brown liquid was further heated, which gave a clear curd. The isolation methods have evolved considerably since then, but the basic principles are still similar; the green juice is extracted from the leaves, the green fraction is removed, and the leaf protein is purified and concentrated as leaf protein concentrate (LPC). The majority of LPC has a biological value between 70 and 83 [4,5], which is comparable to soybean, sunflower seed, and cotton seed meals. According to studies, LPC has a real digestibility between 80 and 90, which is quite comparable to high-quality animal and vegetable proteins [6]. The main issues with storing LPC are microbial breakdown and the onset of rancidity [5].

## 2. Data from the History of Green Biomass as a Protein Source: The Forgotten Early Visions about the Establishment of the Very First Green Protein Biorefinery Factory in Hungary and England: The Green Fodder Mill Concept of Károly (Karl) Ereky

The director of the Science Museum in London, Robert Bud, published that the word “biotechnology” was coined by the mechanical engineer, agricultural economist and minister of nutrition in Hungary, Károly (Karl) Ereky in 1919 [7,8,9,10]. Ereky was a witty, ingenious and creative man. Personally, he was well versed in interdisciplinary happenings and anticipated the achievements as well trends of the development of biotech-based agricultural bio-industry [11,12,13,14]. 

Interestingly, Ereky started his pioneer biotech projects with the establishment of the so-called “*Green Fodder Mill*” factory concept in the spring of 1917. Ereky obtained finely ground green pulp from the leaves of green fodder plants, such as alfalfa, clover, and grasses. This could be wetly fractionated and/or dehydrated to provide feed and food of high protein and vitamin content, the so-called “*green plasma-conserve*” and “*green flour*”, and other commodities [15]. Experimental feeding indicated that the physically processed, stripped from cell walls, green leaves, and young shoots furnished concentrates with a “*complete amino-acid composition*”, “*high-vitamin content*”, and “*100% digestible*” concentrates. Ereky contended that his invention would revolutionize the industrial-scale feeding practices of pig, chicken, and dairy farms. He proved that the use of his green plasma-conserve could save about 40% of feed, especially cereal grain used in pig farms. Moreover, the feeding cycle could be shortened by 20–30%, and egg production capacity was increased to 2.5 times that of laying hen and duck, milk production was improved, and the disease resistance of horses or other domestic animals was optimal [12,13,14]. 

The third classic work of Ereky appeared in Budapest in 1925 under the title: “*The Green Fodder Mill and the Large Scale Industrial Animal Farms*” [12,13,14,16,17]. This book deals with his pioneering research on protein concentrate that could be extracted from the green leaves of plants, otherwise known as leaf protein concentrate (LPC). Its main purpose was the application of the principle of biotechnology in large-scale dairy, milk, and meat or fat-producing farms and “*in the service of the people’s food*” whose theoretical bases had been outlined in the former book “*Biotechnologie*” [15,18,19]. Ereky characterized his process: “*The dried plasma preserve is eminently excellent food for human beings: it contains all the vitamins, inorganic salts and complete albumen-substances in such quantities and of such quality as no other foodstuff does, and moreover easily digestible up to 100%. As in the case of the fresh pulp, the plasma preserve can be rendered more appetizing by the addition of chocolate, sugar, fruit-juice, etc. As a supplementary portion of the human digestive apparatus, the Green Pulper realizes the ancient dream of the vegetarian, and enables man to render himself independent of the consumption of flesh meat*” [16]. 

After many wet fractionation experiments carried out on alfalfa and grasses, some new protocols and different size green pulper machines were developed and patented in Hungary, Britain, and in some other countries between 1924 and 1928 [20,21,22,23,24,25,26]. From 1925, Ereky promoted the huge possibilities of protein production from plant leaves for human consumption [27].

Feeding experiments proved the value of those products of high protein and vitamin content, which means a relatively cheap mass-concentrate, and it was hoped that a new branch of the industry could be founded and developed on the basis of that method. Ereky calculated that the manufacture of 2 to 2.5 tons per hectare of cheap vegetal protein is expected in an industrial system. He stated that the introduction of the “*Ereky-process*” would increase the productivity of the unit agricultural area by a factor of 2–3 times in living weight of pork meat (i.e., 1000 kg ha^−1^), compared with the traditional level (300–600 kg ha^−1^). Similarly, he calculated that a stock of a productive dairy farm fed by a combination with the green protoplasm conservation might attain that of a traditional herd grown on an area of 150,000 ha extensive pasture land but need 12,000 irrigated fields for supplying milk to a population of one million people. He emphasized that this increment was possible only by means of the application of the principles of biotechnology, as the net “level of efficiency” of milk production was increased from 6.5% to 40%. In pig farms, a 7–8 kg dry mixture of cereal was needed for 1 kg of meat live weight, but in the future, a mixture of 3 kg of cereal supplemented with a small amount of green plasma preserve would yield the same live weight [13,14]. 

Ereky’s British connections began in the early 1920s, with correspondence with the Department of Agriculture of the University of Cambridge. Some of this was preserved by the pioneer of the leaf protein research and pioneer of tobacco mosaic virus (TMV) research, Norman Wingate Pirie (1907–1997), who later donated them to The Science Museum in London. Wishing to promote both science and business, in the late spring and summer of 1927, Ereky traveled to Britain and presented his principles and methods to the authorities of the Ministry of Agriculture and Ministry of Health and others from Britain, Canada and Australia [13,14,28,29,30]. 

Practical demonstration activities were also held in Britain with his smaller green pulper. These activities were followed by further pig fattening experiments lasting several months at the Lord Melchett Court (Romsey, Hampshire) organized by Nitram Ltd. (Imperial Chemical Industry, ICI [31]. These were said to prove that “*the nutritive value of one-kilogram young alfalfa juice is equivalent with 2 kg cream-free separated milk*”. The recognition received from an observer in the Ministry of Health was even more enthusiastic, “*the grinding of green stew for human consumption would be even more important than the mash of fodder grass from the point of view of supplying the population*”. Professional opinions emphasized uniformly that with the help of the “*Ereky-process*”, healing products could be developed, which were able “to reorganize the catering tasks of the population of England” [30]. More ambitious, Ereky planned to develop a mass-protein-concentrate process, which could be produced economically in the tropics and could be transported easily to large distances as a green plasma conserve [16]. During his visit to London, the representatives of the Australian government asked him to elaborate on this project. In that country, 4 million sheep were lost annually to temporary drought in semiarid areas, while abundant green fodder grew near the humid seashore [30]. 

On returning to Hungary, Ereky proposed in a March 1928 lecture in the presence of the Hungarian President, Miklós Horthy, that Hungarian agriculture should also be reconstructed by means of the leaf protein concentrate program based on green plasma-preserve produced by his green fodder mill system [30]. It is important to mention that the pioneer papers published by Ereky presented above were not cited by some researchers that followed him in Great Britain and in the USA and approached the wet fractionation process [32,33,34,35,36]. Moreover, Ereky’s British patent was cited by Norman Wingate Pirie in his earliest leaf protein papers published during the Second World War [37,38,39].

## 3. Green Source of Protein

The photosynthesis process occurs mainly in the leaf thylakoid membranes and requires about 70 different proteins [40]. Leaf cytoplasmic protein represents about 20% of leaf protein, while less than 5% and 1.2% of leaf protein are located in mitochondria and cell nuclei, respectively [40]. Kromus et al. [41] identified 250–300 unique proteins and polypeptides in green plant extracts. Photosynthetic protein complexes in the thylakoid membrane represent the majority of the water-insoluble leaf proteins; however, water-soluble leaf proteins also exit [40,42].

Rubisco (ribulose 1,5-bisphosphate carboxylase/oxygenase) is the most prevalent protein in the world, as it is a crucial enzyme for fixing CO_2_ during photosynthesis [40,42]. Rubisco accounts for the majority of the soluble protein component of leaves and up to 50% of their total protein content in C3 plants. A modestly sized enzyme, Rubisco is hexadecameric and has a molecular weight of about 550 kDa. It is a stroma-resident protein and catalyzes the onset of photosynthetic activity. It consists of eight big (55 kDa) and eight small subunits (15 kDa); nevertheless, plants synthesize much more Rubisco in their leaves due to its poor catalytic efficiency [43].

Free amino acids, oligopeptides, and enzymes involved in the synthesis of lipids, proteins, carbohydrates, and other compounds represent the rest part of the soluble leaf proteins [40,44]. Because of this, plant leaves provide a variety of proteins that are typically consumed by cattle that are fed while grazing but may also be utilized for monogastric animal feeding. Green and white proteins are found in different proportions in green leaves, with Rubisco (referred to as fraction I protein) making up the majority of the white proteins [45]. 

**Figure 1:**
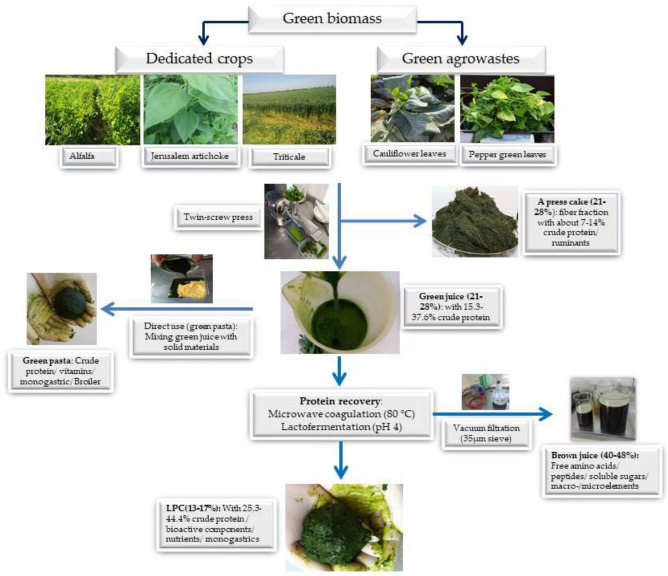
Scheme of basic idea of isolation leaf protein and its related by-products from green biomasses.

The basic principles of leaf protein extraction are illustrated in Figure 1. 

### 3.1. Dedicated Plant Species 

The source of green biomass can be plants grown dedicated to this purpose or green waste as a by-product of vegetable/agricultural crop production. The most commonly used species of crops that can be targeted for green biorefining, including leaf protein concentrates (LPC) preparation, are perennial and annual legumes and grasses (Table 1). At the same time, aquatic crops, such as duckweed, also have great potential [46].

Alfalfa is a regional forage plant that is farmed to a high standard and is GMO-free throughout the European Union [47]. Further, it offers additional ecosystem support services [48], such as improved soil fertility, reduction or avoidance of nitrogen fertilizer use, and pest and disease management [49]. Alfalfa has the ability to provide large yields of dry matter and crude protein per acre in temperate regions [50]. Alfalfa can be used as a source of local protein for a variety of farm animals, which can then produce milk, meat, and eggs for human use.

Due to its availability and balanced amino acid profile, soybean is currently the most popular protein feed. Only 56% of the crude protein utilized in organic farming in Europe is indigenous to the continent [51]. Unlike soy, alfalfa requires less heat and water and can be grown in many parts of the world, including many European regions [52]. 

Jerusalem artichoke (JA) is a well-known crop because of its inulin-rich tubers; notwithstanding, a substantial amount of LPC can be produced from its fresh aerial biomass. The crude protein content in JA-derived LPC was about 33.3% (on a dry basis), and about 53 valuable bioactive phytochemicals were identified, including dimethoxy-tetrahydroxyflavone, dihydroxy-methoxyflavone, hymenoxin, nevadensin, Butein, kukulkanin B, and liqueritigenin [53].

Vetches (*Vicia* spp.) are legumes that are well adapted to the winter season, and therefore they are widely cultivated in West Asia, North Africa, Australia, and Turkey. Vetches can grow on a variety of soil types and are used for a variety of things, including dry matter, silage, and green manure [54]. The vetches spread because of their weak and slender stem. As a result, harvest becomes challenging, and the forage and quality of the plant decline as leaves fall off. In order to prevent spreading, vetch should be mixed with grains before being sowed. Besides increasing the forage yield, the intercropping of vetches with either grasses or cereals provides physical support facilitating its mechanical harvest. Moreover, this mixture might have an additional benefit through increasing leaf protein yield and quality [55] and should be explored. 

Triticale, a hybrid of wheat and rye whose importance is rising globally and which is currently cultivated on more than 400,000 ha, is a perennial plant. When it was first cultivated, its acreage exhibited an upward tendency, had a significant decline in the late 1970s, and has been augmenting since then [56]. Its significance is due in part to its transient character but also due to the important ingredients it contains. Otherwise, triticale has an equivalent value as a plant for making bread and feed. However, in the overall assessment, it is primarily regarded as a grain for fodder. The protein content of triticale might be stated first in terms of its content value.

The crude protein content in the whole triticale plant, fertilized with the standard N-fertilizer (180 kg N ha^−1^), varied from 10.9 to 8.3% when green plants were harvested on 6 June and 6 July, respectively, while plants harvested on 15 August showed a crude protein content of 4.5% in their straw [57]. However, the crude protein percentage increased upon increasing the rate of applied N-fertilizer to 300 kg N ha^−1^. 

According to Jørgensen et al. [58], about 57–74% of the total crude protein in the aboveground green biomass of triticale can be extracted by mechanical pressing into green juice from which 51–63% can be thermally coagulated into an LPC. Similar results of LPC obtained from other green biomass crops were also reported [59,60]. The efficiency of protein extraction or isolation from the green biomasses showed a dependence on plant species rather than the maturity stage/harvest time [61]. On the contrary, Solati et al. [62] found a slight decrease in leaf protein extractability from two grass species; however, this may be attributed to the last harvest at the flowering stage. 

Exogenous N-application positively enhanced the protein extraction efficiency while it lowered the precipitation efficiency. However, this cannot be generalized since the crude protein is usually calculated based on the total N content, and there is a big portion of N that is not a real protein, including nitrate, amino acids, or peptides. These compounds are extractable since they are mostly water-soluble, but they cannot be thermally precipitated [58]. 

Triticale yielded about 730 kg LPC per hectare, which was 40% less compared to grain protein yield. This could be attributed to the low precipitation efficiency of protein in the juice; therefore, there is an urgent need to search for novel technologies to enhance protein recovery [63,64]. 

Nevertheless, the total protein yield of LPC should not be the only ultimate goal. Another important factor that should be considered is the quality of the harvested protein. For instance, grains of triticale and wheat mainly contain storage proteins that are rich in glutamine, glutamate, and proline [65], while leaf protein showed higher contents of limited indispensable amino acids (e.g., lysine, cysteine, and methionine) by 20–25% than grain protein, which makes it suitable protein source for pigs, broilers, and monogastrics as soybean meal.

**Table 1:** Summary of leaf protein yields from different plant species and green agrowastes achieved by varied protein extraction techniques.

Plant Species	Crude Protein Content of Leaf Protein Concentrate (LPC) m/m%	Method of Protein Content Measurement	Protein Isolation Method	Reference
**Dedicated plant species**				
Alfalfa (*Medicago sativa*)	40.3	Kjeldahl	Microwave coagulation	own results (unpublished data)
Alfalfa (*Medicago sativa*)	41.0	Kjeldahl	*Lactobacilus salivarius* fermentation	[66]
Alfalfa (*Medicago sativa*)	40.5 (Green LPC) 32.3 (White LPC)	Kjeldahl/Dumas	Thermal coagulation using two-step heating	[67]
Alfalfa (*Medicago sativa*)	46.0	unknown	Thermal coagulation	[59]
Alfalfa (*Medicago sativa*)	37.8–47.4	Dumas	Acid coagulation	[68]
Jerusalem artichoke (*Helianthus tuberosus*)	33.4	Kjeldahl	Microwave coagulation	[57]
Perennial Rye grass (*Lolium perenne*)	33.9	Kjeldahl	Thermal coagulation	[69]
Perennial Rye grass (*Lolium perenne*, variety; Trocadero and Calvano)	24.5 (Green LPC) 22.8 (White LPC)	Kjeldahl/Dumas	Thermal coagulation using two-step heating	[67]
Ryegrass (*Lolium perenne*)	50.7	unknown	Thermal coagulation	[59]
Red clover (*Trifolium pratense*)	40.0	Kjeldahl	*Lactobacilus salivarius* fermentation	[66]
Red clover (*Trifolium pratense* L., variety; Rajah and Suez)	34.6 (Green LPC) 35.6 (White LPC)	Kjeldahl/Dumas	Thermal coagulation using two-step heating	[67]
White clover (*Trifolium repens* L., variety; Klondike and Silvester)	40.4 (Green LPC) 45.1 (White LPC)	Kjeldahl/Dumas	Thermal coagulation using two-step heating	[67]
Clover and grass mix (*Trifolium pratense and Lolium multiflorum)*	40.0	Kjeldahl	*Lactobacilus salivarius* fermentation	[66]
Clover and grass mix	47.0	unknown	Thermal coagulation	[59]
**Green agrowastes**				
Green pepper (*Capsicum annuum*)	31.2	Kjeldahl	Microwave coagulation	own results (unpublished data)
Green pepper (*Capsicum annuum*)	26.2	Kjeldahl	Lactic acid fermentation	own results (unpublished data)
Horseradish (*Armoracia rusticana*)	25.3	Kjeldahl	Microwave coagulation	own results (unpublished data)
Horseradish (*Armoracia rusticana*)	24.7	Kjeldahl	Lactic acid fermentation	own results (unpublished data)
Forage soy (*Glycine max*)	41.9	Kjeldahl	Microwave coagulation	own results (unpublished data)
Forage soy (*Glycine max*)	37.1	Kjeldahl	Lactic acid fermentation	own results (unpublished data)
Triticale (*×Triticosecale*)	41.0	Kjeldahl	Microwave coagulation	own results (unpublished data)
Triticale (*×Triticosecale*)	34.1	Kjeldahl	Lactic acid fermentation	own results (unpublished data)
Broccoli (*Brassica oleracea, Italica*)	35.3	Kjeldahl	Microwave coagulation	[70]
Broccoli (*Brassica oleracea, Italica*)	39.2	Kjeldahl	Lactic acid fermentation	[70]
Cauliflower (*Brassica oleracea, var. botrytis*)	44.4	Kjeldahl	Microwave coagulation	own results (unpublished data)
Cauliflower (*Brassica oleracea, var. botrytis*)	43.1	Kjeldahl	Lactic acid fermentation	own results (unpublished data)
Brussels sprouts (*Brassica oleracea, var. gemmifera*)	37.4	Kjeldahl	Microwave coagulation	own results (unpublished data)
Brussels sprouts (*Brassica oleracea, var. gemmifera*)	34.4	Kjeldahl	Lactic acid fermentation	own results (unpublished data)
Potato haulm (*Solanum tuberosum*)	45.3	unknown	Thermal coagulation	[71]
Sugar beet *(Beta vulgaris*)	31.3–41.1	Dumas	Thermal coagulation	[60]
Sugar beet *(Beta vulgaris*)	31.1–37.6 (White LPC); 43.6–47.7 (White LPC)	Kjeldahl	Thermal coagulation	[72]
Broccoli (*Brassica oleracea, Italica*)	27.2 (White LPC); 30.4 White LPC	Dumas	Thermal coagulation	[73]
Kale (*Brassica oleracea, var. Sabellica*)	16.7 (White LPC); 30.4 (White LPC)	Dumas	Thermal coagulation	[73]
Cassava (*Manihot esculenta*)	40.4–45.1	Dumas	Thermal coagulation, acid precipitation and spontaneous fermentation	[74]

### 3.2. Green Agrowastes 

Agro-industrial green waste and by-products could serve as readily accessible, affordable, and environmentally friendly sources of plant-based proteins, which can have a marked effect on the decrease of food waste, supporting zero waste initiative and circular economy idea [75]. 

By using a conventional procedure, LPC can be produced from different plant residues, such as aboveground parts, after removing the fruits (Table 1, Table 2 and Table 3). The yield of LPC varied largely according to plant species. For example, carrot top yielded 112 kg ha^−1^, while leaves of cucumber, potato, and tomato produced 1500 kg ha^−1^ with a protein content ranging between 22.5% (carrot) and 50% (potato). The amino acid composition of produced LPC was similar to those obtained from other plant species. 

Proteins differ in their nutritional values, although they are made of similar amino acids. The biological values of potato- and cucumber-derived proteins were 42 and 59%, respectively. During broccoli cultivation, 90% of the above-ground green of the plant is converted to agrowaste, and only 10% enters the food chain. Additionally, it can be used to produce valuable protein concentrate with important nutrients (Table 1, Table 3 and Table 4) through valorization, including fermentation and thermo/microwave coagulation [70,73,76]. Similarly, a side stream of kale is also suitable for green protein concentration purposes [77]. Without being exhaustive, it can be mentioned the Manihot plant (*Manihot esculenta*), which also has great potential. Because of its roots, it is widely cultivated in Africa, Asia, and Latin America. However, the canopy can be used to produce a leaf protein concentrate with a crude protein content of 40–45%, an amino acid profile similar to that of soybean, and tolerable tannin levels (>1% DM) [74].

## 4. Biorefining of Protein from Green Biomass

Due to its nutritional value, high yields, and simplicity of extraction and preparation, LPC may be an effective and sustainable source of proteins for food and feed. However, in order to make LPC more palatable, it must be isolated, purified, and concentrated. 

Several large-scale techniques have been used to extract the protein from green leaves. However, the extraction efficiency of LPC largely varies between 35 and 80%, depending on many factors. 

Agents that disrupt the cellular and subcellular membranes are undoubtedly a determining element in LPC extraction. Moreover, plant species, stage of maturity, plant tissue, the presence of mucilaginous material, postharvest treatment, pH, extractant composition, flotation ratios, extraction time, and temperature [78] also influence this outcome. For example, Solati et al. [62] reported higher crude protein contents in LPC derived from legumes (i.e., white clover, red clover, or alfalfa) than grass species (i.e., ryegrass or tall fescue). Moreover, they documented higher crude protein contents in plant leaves than in plant stems. The extracted amount of LPC did not significantly differ according to the maturity stage of the five investigated plant species.

### 4.1. Extraction of Leaf Protein

The first step in LPC production after collecting the fresh green biomass, mainly leaves and stems, is the mechanical pressing to squeeze out the protein-rich green juice from the fibers [79]. This occurs in two steps: cell walls are first disrupted using different mills or rollers to release intracellular ingredients, mainly soluble proteins and chloroplasts; secondly, the juice is collected by pressing the pulped tissues [79]. However, due to practical limitations, the two steps of green juice extraction could not take place at an industrial scale in a single unit. Therefore, attention was given in early times to developing economically efficient methods to extract green juice [3,80]. 

Screw presses recently showed higher capability in plant-based green juice extraction, as it offers the maceration of the plant cell walls along with desired pressure to squeeze out the juice with an efficient rate of about 60% [81]. Twin-screw extrusion with two propellers on separate shafts with opposite twists revealed higher extraction efficiency of green juice, up to 65% of the inherent fluid found in fresh plant materials with more than 50% of protein precipitation [82,83].

Many researchers suggested re-pressing the press cake after adding an equivalent amount of water because at least 50% of leaf protein is retained in the press cake after the first mechanical pressing [84,85,86]. Knuckles et al. [85] reported a 13% increase in protein recovery after re-pressing water-diluted alfalfa press cake while diluting alfalfa press cake with 5–6% water (on a dry mass basis) increased protein content in green juice by 17% [87]. Similar conclusions were reported by Colas et al. [82] in their study on green juice extraction of alfalfa green biomass using a twin-screw pump at different added water volumes to press cake. Consequently, it can be concluded that the addition of water to press cake, and re-pressing the pressed cake, may be performed if it favors protein recovery. Moreover, chopping plant tissues before pressing could help in releasing the soluble compounds [88].

However, pulping and pressing chopped plant materials in the presence of an alkali solution to bring the pH to 8.0 is recommended rather than pure water. Extraction of leaf protein in an environment with pH ranging between 7.0 and 8.0 was found to improve green juice extraction and protein recovery, where leaf protein becomes more soluble and destruction of cell walls becomes more efficient [89,90]. However, higher pH could bring risks to protein denaturation; therefore, pH should not exceed 8.0. 

Another factor that cannot be ignored during the pressing of green biomass is the temperature of the plant tissues before and during the mechanical pulping. Hanna and Ogden [91] observed that heating the plant materials to above 35 °C considerably reduced the juice extraction and protein recovery, while lower temperatures below 14 °C to 3 °C showed the same effect as the room temperature.

Evidently, the efficiency of juice extraction and protein recovery from fresh plant materials depends on many factors such as pulper device, pH, chopping plant tissues, and temperature. Moreover, the co-extraction of anti-nutritional components should be considered and evaluated. 

### 4.2. Precipitation of Leaf Protein

Isolation of protein from green juice obtained by pressing green biomass is the core step in the production of economically feasible LPC. Consequently, several techniques have been proposed to concentrate leaf protein, which can be classified into three main techniques as follows: (1) differences in solubility (on the basis of distribution coefficient), including salting, organic solvent fractionation, chromatography, crystallization, heating, and centrifugation [92]; (2) gel filtration (on the basis of molecular sizes and shapes), including size-exclusion chromatography and membrane [93]; and (3) isoelectric focusing (on the basis of isoelectric point of proteins), including ion exchange [94].

However, we summarize here the most commonly used techniques for the precipitation of leaf protein from green juices, such as thermal coagulation, fermentation, supercritical CO_2_ extraction, pH extraction, and polyelectrolytes extraction. 

#### 4.2.1. Thermal Coagulation

Recovery of leaf protein from green leaves-derived juice by heat coagulation at a temperature ranges between 60 °C and 95 °C is one of the most commonly applied approaches in the leaf protein precipitation theme [95]. Since Rubisco enzyme represents about 50% of leaf protein [96] and its denaturation temperature is 76.2 °C [97]; therefore, the optimum temperature of leaf protein coagulation is 80 °C [80]. The heat disrupts the protein structure and consequently decreases its water solubility by opening the hydrophobic sites [79]. Proteins found in green juice can be converted into coagulum by heating the green juice to 80 °C for 2–4 min. However, the total coagulum is unpalatable, particularly for human consumption, due to its green color, grassy odor, low water solubility, and digestibility. Consequently, a selective heat coagulation technique was proposed for fractionation of the leaf protein into green and white proteins, which can be directed towards non-ruminants and human consumption, respectively [95]. Green juice is primarily heated to 55 °C to isolate the green protein fraction, then after the supernatant is heated to 80 °C to separate the so-called white protein fraction. The thermal coagulation method of leaf protein is effective; however, it is characterized by many drawbacks such as being energy consuming, the LPC produced has low water solubility, and the properties of protein functional groups could be altered [97]. 

#### 4.2.2. Microwave Coagulation

In addition to other industrial uses, microwave as electromagnetic radiation has recently been used for protein coagulation alone or in combination with conventional thermal coagulation. It is observed that the plant protein coagulum obtained by microwave coagulation is characterized by a coherent colloidal system and dispersed macromolecular structure. The physical consistency of the coagulum is harder, which facilitates separation from the brown liquid during filtration [98,99].

#### 4.2.3. pH Precipitation

Both acidosis and alkalosis can be employed to isolate leaf protein. It is well-known that in conditions of low pH (below 4.5) or high pH (above 8.0), proteins carry positive or negative charges, respectively, which leads to the liberation of several hydrophobic free amino acids, causing a reduction in protein solubility. Usually, acid precipitation of leaf protein is performed by the addition of HCl, while NaOH or ammonia solution is utilized to increase pH to 8.0–8.5. Precipitated protein is subsequently obtained through centrifugation in the form of pellets. For example, acidifying green juice of soybean leaves to pH 3.7 precipitated about 95% of leaf protein [100]. Similarly, proteins in cassava leaves were precipitated by lowering pH to 4.0–5.0, where solubility was at the minimum [101]. Isolation of leaf protein via the pH method may decrease the activity of protease and thus improves the carotene and lutein stability [102]. However, the disadvantages of this method are the loose structure of LPC and the accelerated oxidation of unsaturated fatty acids [64]. 

#### 4.2.4. Acid-Assisted Thermal Coagulation 

Protein can also be denatured by acidosis as a result of changing the overall charges on the amino acids. In this method, the pH of green juice is first lowered by acid addition, followed by heating to isolated leaf proteins. Coagulation of leaf protein by this method is faster than thermal coagulation, and it yields a more compact coagulum compared to the traditional acid precipitation method. Moreover, it produces higher LPC yield in addition to being cost-effective compared to the traditional heating coagulation approach [64]. Moreover, isolated protein by acid-assisted heating method showed higher digestibility and absorption rates. Nevertheless, this method is still energy-consuming and costs more than that of other techniques [64]. 

#### 4.2.5. Microbial Fermentation

Isolation of leaf protein using microbial fermentation has the same basic idea as acid precipitation, where protein solubility largely depends on the pH. However, the main difference between both methods is that instead of adding acids to the green juice, inoculation green juice with acid-producing microbes such as lactic acid bacteria strains (i.e., *Lactobacillus plantarum*, *Pediocuccus cereviseae*, and *Lactobacillus salivarius*) will acidify the juice due to the naturally produced organic acids by these bacteria [64,95]. The reduction in pH of green juice after inoculation with lactic acid bacteria is mainly attributed to the production of lactic acid by the inoculant bacteria. Isolation leaf protein using *L. salivarius* achieved similar results compared to acid precipitation using sulfuric acid [103]. This method is characterized by fewer energy requirements compared to thermal coagulation and less damage to obtained protein. However, it needs a long time and requires specific equipment. 

#### 4.2.6. Protein Precipitation by Flocculants

Flocculants are substances that help tiny particles aggregate, making it simpler to remove them from the liquid phase. Flocculants form large complexes with protein that can be easily isolated from the mixture at room temperature [63]. Several flocculants can be employed in leaf protein recovery, including ionic and non-ionic flocculants [104]. In a study comparing different techniques to isolate leaf proteins in alfalfa green juice, it was found that leaf protein recovery percentages were 42.7%, 42.9%, 45.0%, and 53% with cationic flocculants, acid precipitation (pH 3.5), anionic flocculants, and thermal coagulation, respectively [105]. The exploitation of many flocculants has been examined in green juices obtained from several plant species, including alfalfa, tall fescue, and ryegrass [106]. Lignosulfonates, a by-product of wood pulp production by sulfite pulping characterized by being a hydrophilic and anionic polyelectrolyte polymer, is recently used to precipitate leaf protein from green juice [63]. Compared to thermal coagulation and acid precipitation, lignosulfonates increased LPC yield from green juices of ryegrass, red clover, grass-clover combo, and spinach by 6%, 5%, 7%, and 20%, respectively. The ideal concentration of added lignosulfonates to green juice was 0.6–0.7 g per g protein [63]. 

#### 4.2.7. Supercritical CO_2_ Extraction 

The supercritical CO_2_ extraction for foods and food ingredients was proposed for the first time by Zosel in 1964 [107]. CO_2_ is a solvent that is good for the environment and works well when processing food. The merits that make supercritical CO_2_ a typical extractant are its critical point, which is non-toxic and relatively unreactive. Under physiological and/or highly specialized experimental settings, it has been demonstrated that proteins, amino acids, and amines can interact with carbon dioxide [108]. The impact of supercritical CO_2_ on ribonuclease was investigated as a model protein system in an earlier article [108]. Supercritical CO_2_ may be a more practical and cost-effective way to change how proteins function; the commercial manufacturing of decaffeinated coffee is a good example of this. Additionally, milk and soy proteins that were disseminated in an aqueous solution may be precipitated using CO_2_ as a weak acid [109,110,111]. It is medically safe, inflammable, nontoxic, chemically inert, readily recyclable, and reusable. Furthermore, supercritical CO_2_ has zero surface tension [112], which results in total and quick wetting and permits penetration of complicated structures [113]. When combined with a co-solvent such as ethanol, the supercritical CO_2_ can be utilized to directly alter the composition of commercial whey protein components by extracting lipids and other nonpolar and polar molecules. Depletion or redistribution of nonpolar substances can alter protein structures as well. These elements might present brand-new protein capabilities.

#### 4.2.8. Ultrafiltration 

In order to reduce the energy expenditure in the LPC industry, ceramic membranes have been proposed as an alternative way to isolate leaf protein from green juices as ultrafiltration. This process yields a relatively high LPC amount with protein content ranges between 26.3 and 38.8%, while LPC produced by thermal coagulation exhibited a protein content of 40.1–46.3% [114]. However, ultrafiltration of green juice should be achieved within a short time and at low temperatures to avoid protein hydrolysis. Ref. [97] used a 10 kDa cut-off membrane to separate white protein from alfalfa green juice after eliminating green protein by thermal coagulation. Centrifugation of dissolved alfalfa green juice, which was previously frozen at −25 °C, recovered about 60% of the total N in the juice [115].

## 5. Application of Green Protein Today

### 5.1. State of Art of Green Biomass-Originated Protein in Europe

The production and supply of proteins for the agri-food sector is a recurring issue in the economic policy of the European Union. Within the plant protein utilization market, conventional feed, high-value feed, and food segments can be distinguished. Regarding the feed sector, the EU still relies on imports of protein-rich plant sources, in particular soybeans, which account for almost one-third of all protein used in animal feed [116]. Imports, however, mean vulnerability. Hence, in line with the EU’s “Farm to Fork” strategy, agricultural policy reforms are stimulating local protein feed production and reducing import dependency [116].

**Figure 2:**
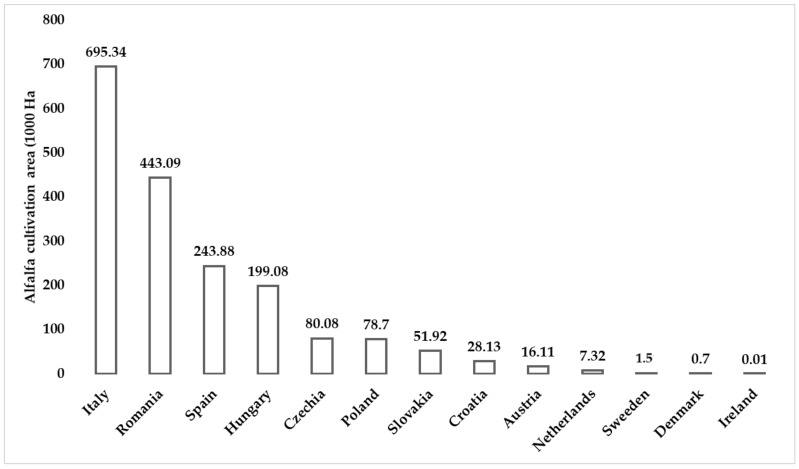
Cultivation area of alfalfa in some EU countries in 2021 from EUROSTAT, 2022 [120].

Considering the agroecological potential of European countries, green biomass proteins offer a real alternative to soy and other seed-based proteins [117]. Green biomass includes grasses; immature cereals and legume crops, such as alfalfa; and clovers as dedicated species in Europe [95]. According to Corona et al., the area of grassland in the European Union (EU) reaches ~16.4 million Ha. Between 10 and 20% of this area could be used for alternative purposes to grazing [59]. While alfalfa is grown on ~2.5 million hectares of pure stands, including legume–grass mixtures, alfalfa is the most widely grown forage legume in almost all of Europe [118]. Currently, the global area of alfalfa is 30 million hectares, of which 25% (7.12 million hectares) is in Europe (Figure 2) [113,119].

Investigating the value and use of green biomass for the production of value-added products and platform molecules is an area of research in Europe with varying intensity from country to country. For instance, Germany, France, the Netherlands, Denmark, and Finland have developed “national protein strategies” to ensure circular and sustainable feed and food supply chains.

In Denmark, there is intensive livestock production. Pork production contributed to 6% of EU-28 production [121,122]. Sustainable pork production also requires an adequate source of feed protein. After cereals, the second most widely grown crop in Denmark is grass and green fodder, with 0.6 million hectares. A well-established selection program has resulted in grass varieties with high yield potential and clover varieties with high protein content, which are being developed for forage purposes [123]. Significant efforts have recently been made in Denmark to develop a green biomass value chain, partly with the aim of reducing dependence on soybean meal imports by using processed green forages.

Based on these, significant efforts have recently been made in Denmark to develop a green biomass value chain, partly using processed green forage, in order to reduce dependence on soybean meal imports [67,68,124]. The research and development have resulted in pilot-scale green biorefineries that demonstrate technical feasibility, such as the decentralized pilot plant at the Agricultural Research Centre in Foulum [125].

Germany is also seeking to make progress in promoting green biorefining by exploiting the surplus grassland biomass. As an example of this, a demonstration green biomass processing facility was planned, directly linked to the already existing green forage drying plant in Selbelang, Havelland. The plant was designed to process 20,000 tonnes of alfalfa and grass biomass yearly, producing platform chemicals and products [126,127].

Grassland makes up nearly 90% of Ireland’s arable land. Among these, perennial rye grass is a widely used fodder crop. Perennial rye grass can be used as green biomass for the production of leaf protein concentrate for pigs by thermal coagulation [69,128]. The protein content of the protein concentrate was 33.9%, and the amounts of lysine and cysteine amino acids were significantly lower than in soybean meal. These results indicated that soybean meal could not be completely substituted by perennial rye grass protein concentrate. However, daily feed intake, feed conversion ratio, and average daily weight gain were better in pigs fed with protein concentrate than in the control.

In the Central-Eastern region of Europe, Hungary has under-utilized potential to develop the green biomass value chain. For instance, Eurostat data indicate that 1,835,000 tonnes of alfalfa were grown on 212,000 ha in 2021. Pioneering work of the “Green Fodder Mill” concept, a process of wet fractionation of green alfalfa biomass, was carried out by Károly (Karl) Ereky in Hungary in the 1920s [16,17,31,37]. In the 1960s, the VEPEX (Vegetable Protein Extract) program was launched, based on a technology to produce high biological value, fiber-free feed from green plants. A steam injection coagulation-based technology developed and patented was applied on an industrial scale in Tamasi and Ács (Hungary) [129]. In the early 2000s, green biorefining gained new momentum. A semi-industrial demonstration plant was built by the Tedej Co. Ltd. (Hajdúnánás-Tedej, Hungary) in cooperation with the University of Debrecen, based mainly on green biomass from lucerne. The plant was capable of producing 200 L h^−1^ of green juice, from which ~520 kg h^−1^ of leaf protein concentrate (36–46 m m^−1^% crude protein) could be produced by combined heat and microwave coagulation [130]. In 2019, an industrial size green protein biorefinery factory was constructed in Hungary called PROTEOMILL, which technology has also been patented [98]. Its actual production capacity is 4000 L of green juice per hour. The fresh alfalfa green biomass is harvested from a 120–150 ha plantation belonging to the PROTEOMILL factory (Tedej Co. Ltd., Hajdúnánás-Tedej, Hungary).

France is one of the major alfalfa producers in the EU and exports the crop mainly in dried form to different countries. Along with it, they use well-known technologies such as fractionation at large scale [119]. As mentioned above, plant leaf protein can be used not only as feed but also as food. In Europe since the 1960s, the increasing proportion of plant-based dietary proteins relative to animal proteins in both consumption and production can be observed. A shift to a plant-based diet to meet the UN Sustainable Development Goals and the Paris Agreement presents the Planetary Health Diet as a benchmark for a healthy and sustainable diet for a growing population [131]. In 1993, at the suggestion of France Luzerne, the APEF (Association pour la Promotion des Extraits Foliaires en Nutrition, Paris) launched a program for the use of alfalfa leaf protein concentrate for human consumption [132]. A pilot plant was launched in 1997 with the participation of five companies, including Alfa-Laval. Then, with the approval of the EU, the FRALUPRO (“Fractionation of alfalfa juice lo to create a functional and functional protein ingredient for the food and non-food industry”) program was launched, with the design of the first 1200 t year^−1^ capacity so-called Rubisco protein plant. Alfalfa leaf protein-xanthophyll concentrate (APC), which contains more than 50% total protein and 1200–2200 mg dm^−3^ xanthophyll, is approved by EFSA as a food supplement [133,134].

### 5.2. State of Art of Green Biomass-Originated Protein in America

The urge to shift from animal to plant-based foods observed in Europe is also experienced in America [135,136,137,138,139]. In the globalized world market, other continents are expected to face similar challenges. This is due to an increasing world population [140,141] and to meet a demand for lower energy and labor inputs to food production. Success in this matter means sustaining our feeding needs and consists of an intricate web involving psychological [142], political [136], environmental [143,144], cultural, economic, and personal issues [136,139,141,142,145]. 

The international trade market is either an active or passive player, suffering and leading the outcomes that run through agricultural and livestock products [136,140,143]. For instance, events such as the COVID-19 pandemic [146]; others such as the Russia–Ukraine conflict; and shades of political, cultural, and ideological beliefs may particularly influence our capacity to produce and consume food. Consequently, caution and consciousness are essential for success, locally and worldwide. 

Plant- and meat-based products have always been part of the human diet [147]. Further, we are constantly faced with new food ingredients and the possible reuse of agricultural byproducts that can minimize waste and pollution [148]. A compiled guidance for reliable and environment-friendly feeding habits is observed [136,140,149], and it includes the change to plant-based food protein, new food ingredients, and the due regulation of food production [135,136,141]. The use of plant varieties that are less demanding on energy, minerals, and other inputs while, ideally, presenting more nutritive, productive potential, and increased acceptance is encouraged. This comprises the continuous efforts of plant breeding and dealing with climate change and the decrease in water resources that, despite imperative, are not covered here. 

There are commercial perspectives and patent interest in plant-based protein food (Table 5). Nevertheless, it seems that the interest in this market overrules the attention to nutritional, healthy, and other science-related issues with plant-based foods. It appears to be, at least, a delay in the compass among the information and actions in the trade sector, research initiatives, and consumer preferences. Detailed market analysis on plant-based foods and protein and products are available (Table 5), whereas information on the protein quality and amino acid composition may be recent or incipient [149]. Despite the possible bias in the World Wide Web information and the need for datamining expertise, we face an evolving area of merchandise and advertisement on plant-based foods. 

The focus on the market and advertisement on plant-based food and protein is surprising! This leads us to question “the state of market of green biomass and protein” in addition to the “state of art of green biomass originated protein in America”. We observe attractive “market polls” and promising niche of plant-based protein (Table 5). This covers conflicting information on the internet approaching opinions, polls, markets, etc. This will easily confuse the reader and the consumer, so we are compelled to ask: what is the role of science in this trend? Scientific articles cover objectives from the opinion, psychological and healthy issues of the consumer to the approach of protein extraction protocols. 

Patents dealing with the processing of plant-based foods (Table 5) as the endorsement of the positive contribution of plant-based diets to human health [150,151] are available. This figure ought to be higher since Arbach et al. [152], for instance, found 113 patent applications related to plant-based beverages. Scientific research may provide new and improved plant varieties and species through selection, breeding, and biotechnological tools that afford biomass and protein as food for humans [148,153,154,155,156]. 

Despite globalized and scattered information, plant-based foods and plant (and other sources of) protein [135,136,138] are deemed to be at the forefront of our interest. There are recent approaches to the transition to plant-based protein [136,139,145,157,158], signaling efforts of the scientific community to keep up with this trend. 

A bibliometric analysis on “the disposition of reducing meat consumption” was conducted by gathering information from 1994 to 2020 [158]. According to the authors, none of the consulted articles of the eight most productive journals focused on marketing. This was attributed to the lack of interest in the subject, although consumer behavior regarding reducing meat consumption was identified as a gap and relevant topic for marketing research. Although the reduction in animal-based protein was identified earlier, the outcomes from a transition to plant-based protein alternatives have been explored only recently. Sha and Xiong [159] also recognized an increase in scientific publications in the last decade approaching meat alternatives and analogs. 

The need for a filter and northerning the information completes the canvas of our state-of-the-art of green mass protein production in America. In this brief, we sought articles and information derived or associated with data gathered in the American continent. The adoption of plant-based protein is expected for several reasons, as pointed out in Moreira et al.’s [158] analysis, focused on North American, European, and Oceania countries. Plant protein and plant-based meat are increasing, concomitantly with product diversity, regardless of the lack of understanding of the consumer view [157]. This intriguing observation let out of the block a missing engine that is propelling this system, and it is based not only on the consumer. 

Marinangeli et al. [160] compiled the importance, advantages, and market of plant-based protein, addressing the need for a regulatory landscape and formulation guidance for these innovative products. For instance, the requirement for standards for essential amino acids and digestibility approaches of these plant-based products according to life stage and health are needed. Opposingly, the choice of beef and plant-based foods were not affected by the nutrition facts panels or ingredient lists [161]. There are differences in the willingness for the preference and nutritional perception of these foods based on the assortment of general consumers and nutrition professionals [157]. This highlights the importance of information as a promotor for the consumption and adjustments of the composition [156], organoleptic properties, and other traits [135,159] of plant-based foods and proteins. 

The negative relationship between predisposition changes in behavior for the adoption of a sustainable diet and the lack of information on plant-based diets [142] pinches objectives for future research. Focus themes such as refining the positive health effects, affordable products, improving behavior, and the disclosure of amino acid and digestibility standards of the plant-based proteins are other research inputs. The belief that these products contribute to good health [150,151] and are friendlier to the environment [136] may lead consumers to be increasingly amenable to plant-based diets and plant-based meat alternatives. Accordingly, economic modeling suggests positive effects of the adoption of plant-based beef alternatives such as reduced greenhouse gas emissions, reduced carbon footprint, and exports of agriculture products. On the other hand, it may result in the disruption of cattle and beef processing sectors throughout the agriculture economy, which will face a complex ethical and political transition [145]. 

Despite the origin (species, plant sample, and the use of raw or processed samples) of plant-based protein products, there is a niche for the development and commerce of alternative foods derived from plant biomass. The innovative capacity and speed of product development of the rising food companies contribute to the landscape. In Latin America, there are intents and actions recognized as a laboratory and a showcase for inhibiting the consumption of (ultra) processed food [162]. Nevertheless, there is an increase in the consumption of industrialized food and other sources of animal protein [140] due to the increase in population income and cheaper nutritional sources [136,162,163]. 

Consumers are recently challenged with new plant-based products that illustrate the innovation capacity and the speed of action expected from South America’s entrepreneurs. “Future Farm” brand, a plant-based meat company in Brazilian and abroad markets. It is also working on and planning the release of plant-based milk, cheese, and chicken products. Chile’s NotCo is an important food company founded in 2015 that is investing in plant-based lactic products and is considered one of the fastest-growing Food Companies in LATAM. There are other South American brands, such as “The Live Green Co.” and “The New Butchers”, and North American brands, such as “Beyond Meat” [135] and others that are investing in plant-based food products (Table 5). 

De Marchi et al. [139] reported similar characteristics of meat-based (MBB) and plant-based burgers (PBB), such as color, pH, gross composition, total fatty acid profile, and protein, although there were more carbohydrates and fiber content (PBB) and significant differences in amino acids, polyunsaturated fatty acids, cholesterol, and minerals. Soy and pea protein and beet are listed as ingredients for the plant-based protein PBB. Other interesting plant-based products are beverages enriched with plant proteins. Despite Arbach et al.’s [152] review, which basically reports the use of seed-protein in these beverages that benefited from lower prices and environmental damage, there is also an application for green juices and plant biomass [164]. 

It seems that seed-protein-based ingredients are the first option, although there is an open window for other plant-biomass ingredients. There are efforts to use green juice from plant biomass as possible raw material for plant-based beverages [165]. There are some promising initiatives of plants that should be revisited as protein sources, such as the use of cassava leaves [166] that, probably to HCN content in leaf tissues, seem to be abandoned. Alternative ingredients, either non-seed or non-animal origin, for these new food and non-food products are also available [159]. The use of ingredients from other plant species may contribute to equalizing meat-based and plant-based product characteristics. Biofabric products are alternatives that can contribute to an increased bioaccessibility and bioavailability of green plant biomass [137,138,148,164,167]. This includes the use of biofabrics to extract protein from plant biomass [148,164]. 

Regardless of pioneer initiatives [139,156,167], a further contribution from academic research could be the development of protein from green biomass as a potential alternative to seed-based protein. It should be noted that there are other applications for plant-based materials, such as fuel production [164,168,169], that are not discussed here. The key to successful application lies in the processing possibilities and in the applied plant species/varieties [138,148]. 

In summary, there are gaps in the chains among commerce, consumers, industry, and producer, and the development and research on plant-based protein products ought to be fulfilled to achieve the benefits of these food alternatives. The high technology, investments, and productivity of countries such as the US, the tradition in agribusiness, the territory availability of countries such as Brazil, and entrepreneur initiatives and companies, indicate that protein and other products derived from plant biomass is a fertile areas in American countries.

### 5.3. State of Art of Green Biomass-Originated Protein in Africa

Poverty and food shortage, especially a protein-rich diet, are the driving factors of malnutrition and its detrimental consequences in Africa [170]. Green biorefinery may serve as a substituent for food insecurity as it helps to fulfill food and feed needs for Africa. Processing green biomass into acceptable products for direct human consumption may have nutritional, economic, and environmental merits [171]. 

According to several researches being conducted, there are some particular plants to use as biomass for a sustainable protein supply in Africa. The most prominent ones are *Moringa oleifera*, *Manihot esculenta*, *Glyricidia sepium,* and *Leucaena leucocephala*. 

*Moringa oleifera* is a fast-growing tree from the family of Moringaceae, which is highly drought-resistant. It has a high profile of nutritional composition and is most widely cultivated in Africa. *Moringa oleifera* leaf protein concentrate is a promising source of protein for most of the developing countries in Africa. LPC obtained from *Moringa oleifera* is nutritionally valuable [170]. 

According to Sodamade’s research [170], *Moringa oleifera*’s leaf protein concentrate’s moisture content is 9.00 mg 100 g^−1^, ash content is 6.00 mg 100 g^−1^, crude fat is 2.43 mg 100 g^−1^, crude fiber is 5.43 mg 100 g^−1^, carbohydrate content is 38.21 mg 100 g^−1^, and crude protein content is 39.13 mg 100 g^−1^. This remarkable amount of crude protein in the plant means that *Moringa oleifera* leaf protein concentrate may be evaluated as nutritionally valuable and a healthy ingredient to improve protein needed in the human diet and animal feed. Its functional properties, such as water absorption capacity, fat absorption capacity, emulsion capacity, and foaming stability, are also significant [170]. 

*Manihot esculenta*, also known as cassava or manioc, is a tuberous edible plant of the Euphorbiaceae family. Since cassava flour, bread, tapioca, an alcoholic beverage, and laundry starch are derived from its tuberous roots, it is cultivated in many plantations in Africa. Moreover, 250 million Africans make use of the starchy root crop *Manihot esculenta* as an important part of their diet [172].

Cassava plays an important role in agriculture, especially in sub-Saharan African countries. Cassava leaves are consumed as a major source of dietary protein for all of Central Africa, most of East Africa, and even some parts of West Africa [173]. 

Both the leaves and roots of the cassava plant are nutritionally valuable, and they offer the potential as a feed source. The root of cassava is mostly a carbohydrate resource that contains 60–65% moisture, 20–31% carbohydrate, and 1–2% crude protein. It contains a relatively low content of vitamins and minerals [174].

Cassava leaves are potential biomass that is affluent in protein with a balanced content of amino acids. Thus the leaves represent valuable biomass for the extraction of proteins. According to Gundersen’s study [74], between 21% and 26% (*w*/*w*) of leaf crude protein can be recovered in the leaf protein concentrates. After the drying process, the product contains 40–45% crude protein with an amino acid notable profile that can be compared with soybean. Its level of tannins is tolerable in the case of animal feed purposes [74].

The cassava leaf protein concentrate’s (LPC) amino acid profile is fairly similar for the starting leaf material and the press cake. Except for aspartic and glutamic acid, for most amino acids, the content is barely lower in the produced green juice. In research that compared the amino acids profiles of cassava LPCs obtained by heat coagulation and acid precipitation with the soybean reference, it was found that cassava LPCs’ content of methionine, leucine, and valine is higher than the soybean reference [74].

On the other hand, Cassava has some antinutritional elements. Cassava leaves’ cyanogenic glucosides potential is 5 to 20 times higher than the cyanogenic potential of its roots. Although there is a risk of intoxication held by the consumption of cassava leaves, during processing, the risk is decreased due to the capacity of the leaves to release cyanogens quickly [173]. The releasable HCN amount existing in the dried protein product is around 150–250 ppm. However, this amount is still higher than 10–50 ppm which is considered safe for food and feed purposes by the food safety authorities [74]. 

In sub-Saharan Africa, *Glyricidia sepium* and *Leucaena leucocephala* are also notable plants for biorefinery purposes. They have foliage production ability for all-year-round. Moreover, they are rich in protein, minerals, and vitamins [175].

*Gliricidia sepium* is a tropical forage plant from the Fabaceae family. Its leaves are considered to contain high protein content and are suitable for producing protein-rich forage with its high nutritive value. It is a plant that shows a wide distribution and variation in productivity. *Leucaena leucocephala* is a fast-growing evergreen plant from the Fabaceae family. Its young leaves and seeds may be used as a vegetable in human nutrition [176].

In sub-Saharan Africa, *Glyricidia sepium* and *Leucaena leucocephala* leaves mount up all year round. The leaves have rich protein, minerals, vitamins, and amino acid content. Therefore, *Glyricidia sepium* and *Leucaena leucocephala* are convenient for producing leaf protein concentrates. According to the research carried out by Agbede and Aletor [176], *Leucaena leucocephala* leaves involve lower crude protein and higher crude fiber than *Glyricidia sepium* leaves, but their ash values are equal. Their crude protein in the LPCs showed amino acids with a good balance. Their LPCs have similar amino acid profiles, but the values of Glyricidia LPC (G. LPC) are generally a little higher than Leucaena LPC (L. LPC, except for proline and methionine. Some of the amino acid compositions (g 100 g^−1^ sample) of leaf protein concentrate that were measured during their research are lysine 5.99 in L. LPC and 6.60 in G. LPC; histidine 2.11 in L. LPC and 2.51 in G. LPC; arginine 5.54 in L. LPC and 6.30 in G. LPC; Threonine 4.61 in L. LPC and 5.08 in G. LPC; methionine 2.25 in L. LPC and 2.05 in G. LPC. Due to this high nutritional amino acid concentration, Glyricidia and Leucaena LPCs are comparable with whole egg amino acids profile [176].

In conclusion, with its relatively high protein content and excellent amino acid profile, Glyricidia or Leucaena LPC may be a successful substitute for the soybean, which is a more expensive protein source and a sustainable alternative for supplying affordable food in Africa [175].

## Figures and Tables

**Table 2:** Amino acid composition (m/m%) of leaf protein concentrates from selected green biomass and seed-based protein sources bypre-coulmn derivatization method of UHPLC (own measurements).

Amino Acids	Alfalfa (LPC)	Alfalfa (Green Juice)	Jerusalem Artichoke (LPC-MW)	Green Pepper (LPC-MW)	Green Soy (LPC-MW)	Cauliflower (LPC-MW)	Soy (Seed Extracted)	Triticale (Green Juice)	Triticale (LPC-Microwave)	Triticale (LPC-Lactic Acid)
ASP	5.22	4.33	4.24	2.86	4.29	5.23	5.64	2.72	3.84	2.68
THR	2.44	1.76	1.88	1.27	1.97	2.73	1.93	1.11	1.59	1.62
SER	2.34	1.67	1.91	1.30	2.10	2.40	2.43	1.19	1.69	1.72
GLU	5.27	4.01	4.09	3.34	4.57	5.95	8.76	2.68	3.55	3.57
PRO	2.10	1.44	2.04	1.53	2.10	2.60	2.34	1.24	1.78	1.78
GLY	2.55	1.79	1.70	1.68	2.23	2.71	2.06	1.23	2.07	2.06
ALA	2.89	2.02	1.94	1.79	2.66	2.52	1.99	1.60	2.43	2.38
CYS	0.11	0.11	0.22	0.15	0.15	0.77	0.20	0.12	0.20	0.20
VAL	2.73	1.96	1.47	1.77	2.25	1.93	2.27	1.42	2.04	2.05
MET	0.25	0.21	0.61	0.32	0.67	1.11	0.31	0.29	0.41	0.42
ILE	2.20	1.58	1.25	1.41	1.71	1.99	2.14	0.97	1.47	1.47
LEU	4.37	2.96	3.21	2.74	3.84	3.83	3.70	2.03	3.26	3.26
TYR	1.53	1.11	1.35	1.29	1.82	2.33	1.50	0.97	1.51	1.53
PHE	2.74	1.83	1.99	1.73	2.42	3.43	2.44	1.31	2.24	2.26
HIS	1.11	0.74	1.28	0.67	0.92	1.03	2.47	0.52	0.80	0.82
LYS	4.15	2.33	1.90	1.94	2.76	1.94	3.92	1.44	1.91	1.89
ARG	2.10	0.22	2.07	1.72	2.67	2.78	2.61	1.54	2.18	2.12

**Table 3:** Macro- and microelements composition (mg kg^−1^) of selected green biomass and seed-based protein sources based on own ICP-OES measurements.

Element	Alfalfa (Green Juice)	Alfalfa (LPC)	Broccoli (Green Juice)	Soy Seed (Extracted)
Mo	7.9	4.1	1.4	1.9
Cu	11.4	23.9	2.9	13.0
Ba	6.3	12.4	6.1	20.6
B	32.8	20.2	13.5	26.2
Zn	33.6	38.2	29.8	36.3
Mn	28.4	48.4	47.7	32.3
Sr	69.0	65.9	183.0	7.4
Al	92.0	145.8	68.8	36.7
Fe	145.3	315.9	97.2	88.4
Na	411.9	91.0	5116.2	7.1
Mg	3845	2149	8759	2268
S	4310	4682	13,653	2283
P	5457	7290	3308	5721
Ca	16,050	16,017	16,266	1862
K	40,020	21,245	20,770	14,654

**Table 4:** Qualitative phytochemical analysis of selected green biomass based on own UHPLC-ESI-MS measurements.

LPC-Alfalfa		LPC-Broccoli		LPC-JA	
Chemical Name	Chemical Formula	Chemical Name	Chemical Formula	Chemical Name	Chemical Formula
4′.5.7-Trihydroxyflavanone (Naringenin)	C_15_H_12_O_5_	Isoliquiritigenin (2′,4,4′-trihydroxychalcone)	C_15_H_12_O_4_	γ-Aminobutyric acid	C_4_H_9_NO_2_
4′.5.7-Trihydroxyflavanone 6.8-C-glucoside	C_27_H_32_O_15_	Quercetin (3,3′,4′,5,7-Pentahydroxyflavone)	C_15_H_10_O_7_	Quinic acid	C_7_H_12_O_6_
4’.7-Dihydroxyflavanone	C_15_H_12_O_4_	Quercetin-*O*-hexoside-*O*-hexosylhexoside isomer 1	C_33_H_40_O_22_	Betaine (Trimethylglycine)	C_5_H_11_NO_2_
Quercetin	C_15_H_10_O_7_	Quercetin-3-*O*-[caffeoyl-(→2)-glucosyl-(1→2)-glucoside]-7-*O*-glucoside	C_42_H_46_O_25_	Malic acid	C_4_H_6_O_5_
Quercetin-3-*O*-glucoside	C_21_H_20_O_12_	Quercetin-*O*-(sinapoyl)hexosylhexoside-*O*-hexoside	C_44_H_50_O_26_	Nicotinic acid (Niacin)	C_6_H_5_NO_2_
Apigenin-4′-*O*-glucuronide-7-*O*-[glucuronyl-(1→2)-glucuronide]	C_33_H_34_O_23_	Quercetin-3-*O*-[feruloyl-(→2)-glucosyl-(1→2)-glucoside]-7-*O*-glucoside	C_43_H_48_O_25_	Citric acid	C_6_H_8_O_7_
Apigenin-*O*-glucoside-*O*-glucuronide	C_27_H_28_O_16_	Quercetin-*O*-hexoside-*O*-hexosylhexoside isomer 2	C_33_H_40_O_22_	Neochlorogenic acid (5-*O*-Caffeoylquinic acid)	C_16_H_18_O_9_
Apigenin-7-*O*-[feruloyl-(→2)-[glucuronyl-(1→3)]-glucuronyl-(1→2)]glucuronide	C_43_H_42_O_26_	Quercetin-*O*-hexosylhexoside isomer 1	C_27_H_30_O_17_	Salicylic acid-2-*O*-glucoside	C_13_H_16_O_8_
Apigenin-4′-*O*-glucuronide-7-*O*-[feruloyl-(→2)-glucuronyl-(1→2)-glucuronide]	C_43_H_42_O_26_	Quercetin-di-*O*-hexoside	C_27_H_30_O_17_	Chlorogenic acid (3-*O*-Caffeoylquinic acid)	C_16_H_18_O_9_
Apigenin-7-*O*-glucuronide	C_21_H_28_O_11_	Quercetin-*O*-hexosylhexoside isomer 2	C_27_H_30_O_17_	Chryptochlorogenic acid (4-*O*-Caffeoylquinic acid)	C_16_H_18_O_9_
Apigenin (4′.5.7-Trihydroxyflavone)	C_15_H_10_O_5_	Quercetin-3-*O*-glucoside (Isoquercitrin)	C_21_H_20_O_12_	4-*O*-(4-Coumaroyl) quinic acid	C_16_H_18_O_8_
Chrysoeriol-7-*O*-glucuronide	C_22_H_20_O_12_	Kaempferol (3,4′,5,7-Tetrahydroxyflavone)	C_15_H_10_O_6_	Vanillin (4-Hydroxy-3-methoxybenzaldehyde)	C_8_H_8_O_3_
Chrysoeriol (3′-Methoxy-4′.5.7-trihydroxyflavone)	C_16_H_12_O_6_	Kaempferol-*O*-hexoside-*O*-hexosylhexoside	C_33_H_40_O_21_	5-*O*-(4-Coumaroyl)quinic acid	C_16_H_18_O_8_
Chrysoeriol-glucuronyl-glucuronide	C_28_H_28_O_18_	Kaempferol-7-*O*-glucoside-3-*O*-sophoroside	C_3_3H_40_O_21_	Indole-3-acetic acid	C_10_H_9_NO_2_
Genkwanin (4′,5-Dihydroxy-7-methoxyflavone)	C_16_H_12_O_5_	Kaempferol-*O*-(caffeoyl)hexosylhexoside-*O*-hexoside	C_42_H_46_O_24_	4-*O*-(4-Coumaroyl)quinic acid cis isomer	C_16_H_18_O_8_
Luteolin-7-*O*-glucuronide	C_21_H_18_O_12_	Kaempferol-3-*O*-[caffeoyl-(→2)-glucosyl-(1→2)-glucoside]-7-O-glucoside	C_42_H_46_O_24_	Isoscopoletin (6-Hydroxy-7-methoxycoumarin)	C_10_H_8_O_4_
Luteolin (3′.4′.5.7-Tetrahydroxyflavone)	C_15_H_10_O_6_	Kaempferol-*O*-(caffeoyl)hexosylhexoside-*O*-hexosylhexoside	C_48_H_56_O_29_	5-*O*-Feruloylquinic acid	C_17_H_20_O_9_
Tricin-7-*O*-glucuronide	C_23_H_22_O_13_	Kaempferol-3-*O*-[caffeoyl-(→2)-glucosyl-(1→2)-glucoside]-7-*O*-[glucosyl-(1→4)-glucoside]	C_48_H_56_O_29_	Riboflavin	C_17_H_20_N_4_O_6_
Tricin-7-*O*-[feruloyl-(→2)-glucuronyl-(1→2)-glucuronide]	C_39_H_38_O_22_	Kaempferol-3-*O*-[sinapoyl-(→2)-glucosyl-(1→2)-glucoside]-7-*O*-glucoside	C_44_H_50_O_25_	Scopoletin (7-Hydroxy-6-methoxycoumarin)	C_10_H_8_O_4_
Tricin (3′.5′-Dimethoxy-4′.5.7-trihydroxyflavone)	C_17_H_14_O_7_	Kaempferol-3-*O*-[sinapoyl-(→2)-glucosyl-(1→2)-glucoside]-7-*O*-[glucosyl-(1→4-)glucoside]	C_50_H_60_O_30_	Azelaamic acid (9-Amino-9-oxononanoic acid)	C_9_H_17_NO_3_
Tricin-*O*-hexoside	C_22_H_24_O_12_	Kaempferol-3-*O*-[feruloyl-(→2)-glucosyl-(1→2)-glucoside]-7-*O*-glucoside	C_43_H_48_O_24_	6-Methylcoumarin	C_10_H_8_O_2_
4′.7-Dihydroxyflavone	C_15_H_10_O_4_	Kaempferol-3-*O*-[feruloyl-(→2)-glucosyl-(1→2)-glucoside]-7-*O*-[glucosyl-(1→4)-glucoside]	C_49_H_58_O_29_	5-*O*-(4-Coumaroyl)quinic acid cis isomer	C_16_H_18_O_8_
Methoxy-tetrahydroxyflavone	C_16_H_12_O_7_	Kaempferol-*O*-[p-coumaroyl-(→2)-glucosyl-(1→2)-glucoside]-7-*O*-glucoside	C_42_H_46_O_23_	Indole-4-carbaldehyde	C_9_H_7_NO
Dimethoxy-hydroxyflavone	C_17_H_14_O_5_	Kaempferol-3,7-di-*O*-glucoside (Paeonoside)	C_27_H_30_O_16_	Fraxidin or Isofraxidin	C_11_H_10_O_5_
3′-Methoxy-4′.5.5′.7-tetrahydroxyflavone-7-*O*-glucuronide	C_22_H_20_O_13_	Kaempferol-*O*-(sinapoyl)hexosylhexoside-*O*-(sinapoyl)hexoside	C_55_H_60_O_29_	Loliolide	C_11_H_16_O_3_
Apigenin-8-C-glucoside-6-C-xyloside	C_26_H_28_O_14_	Kaempferol-di-*O*-hexoside	C_27_H_30_O_16_	4-Hydroxy-3-methoxycinnamaldehyde (Coniferyl aldehyde)	C_10_H_10_O_3_
Apigenin-6-C-glucoside-8-C-xyloside	C_26_H_28_O_14_	Kaempferol-*O*-(caffeoyl)hexosylhexoside	C_36_H_36_O_19_	7-Deoxyloganic acid isomer	C_16_H_24_O_9_
Alfalone (4′.7-Dimethoxy-6-hydroxyisoflavone)	C_17_H_14_O_5_	Kaempferol-*O*-(sinapoyl)hexosylhexoside	C_38_H_40_O_20_	Di-*O*-caffeoylquinic acid isomer 1	C_25_H_24_O_12_
Formononetin (7-Hydroxy-4′-methoxyisoflavone)	C_16_H_12_O_4_	Kaempferol-7-*O*-sophoroside	C_27_H_30_O_16_	Di-*O*-caffeoylquinic acid isomer 2	C_25_H_24_O_12_
Ononin (Formononetin 7-*O*-glucoside)	C_22_H_22_O_9_	Kaempferol-*O*-(feruloyl)hexosylhexoside	C_37_H_38_O_19_	Salvianolic acid derivative isomer 1	C_27_H_22_O_12_
Biochanin A (4′-Methylgenistein)	C_16_H_12_O_5_	Kaempferol-*O*-(4-coumaroyl)hexosylhexoside	C_36_H_36_O_18_	Butein (2′,3,4,4′-Tetrahydroxychalcone)	C_15_H_12_O_5_
Isoliquiritigenin (2′,4,4′-trihydroxychalcone)	C_15_H_12_O_4_	Kaempferol-*O*-(disinapoyl)hexosylhexosylhexoside-*O*-hexoside	C_61_H_70_O_34_	Quercetin-3-*O*-glucuronide	C_21_H_18_O_13_
Medicagenic acid	C_30_H_46_O_6_	Kaempferol-*O*-hexosylhexoside	C_27_H_30_O_16_	Isoquercitrin (Hirsutrin, Quercetin-3-*O*-glucoside)	C_21_H_20_O_12_
Medicagenic acid 28-*O*-[xylosyl-(1→4)-rhamnosyl-(1→2)-arabinosyl]ester	C_46_H_72_O_18_	Kaempferol-3-*O*-glucoside (Astragalin)	C_21_H_20_O_11_	Chrysoeriol-*O*-glucoside	C_22_H_22_O_11_
Medicoside H (Medicagenic acid 3-*O*-glucosyl-28-*O*-[rhamnosyl-(1→2)-arabinosyl]ester)	C_47_H_74_O_19_	Isorhamnetin-*O*-hexosylhexoside	C_28_H_32_O_17_	Salvianolic acid derivative isomer 2	C_27_H_22_O_12_
Medicoside G (Medicagenic acid 3,28-di-*O*-glucoside)	C_42_H_66_O_16_	Isorhamnetin-3-*O*-glucoside	C_22_H_22_O_12_	Di-*O*-caffeoylquinic acid isomer 3	C_25_H_24_O_12_
Medicagenic acid 3-*O*-glucuronide-28-*O*-[xylosyl-(1→4)-rhamnosyl-(1→2)-arabinosyl]ester	C_52_H_80_O_24_	Isorhamnetin-7-*O*-glucoside-3-*O*-sophoroside (Brassicoside)	C_34_H_42_O_22_	Azelaic acid	C_9_H_16_O_4_
Medicagenic acid rhamnosyl-pentosyl-glucuronide	C_47_H_72_O_20_	4′.7-Dihydroxyflavanone (Liquiritigenin)	C_15_H_12_O_4_	Kaempferol-3-*O*-glucuronide	C_21_H_18_O_12_
Medicoside J (Medicagenic acid 3-*O*-glucosyl-28-*O*-[xylosyl-(1→4)-rhamnosyl-(1→2)-arabinosyl]ester)	C_52_H_82_O_23_	4′,5,7-Trihydroxyflavanone (Naringenin)	C_15_H_12_O_5_	Apigenin-*O*-malonylglucoside	C_24_H_22_O_13_
Soyasapogenol B rhamnosyl-hexosyl-glucuronide	C_48_H_78_O_18_	Apigenin (4′,5,7-Trihydroxyflavone)	C_15_H_10_O_5_	Astragalin (Kaempferol-3-*O*-glucoside)	C_21_H_20_O_11_
Soyasapogenol B rhamnosyl-pentosyl-glucuronide	C_47_H_76_O_17_	Apigenin-7-*O*-glucuronide	C_21_H_28_O_11_	Isorhamnetin-3-*O*-glucoside	C_22_H_22_O_12_
Azukisaponin II	C_42_H_68_O_14_	Luteolin (3′.4′.5.7-Tetrahydroxyflavone)	C_15_H_10_O_6_	Kukulkanin B (2′,4′,4-Trihydroxy-3′-methoxyxchalcone)	C_16_H_14_O_5_
**Unknown saponins**		Neochlorogenic acid (5-*O*-Caffeoylquinic acid)	C_16_H_18_O_9_	Isorhamnetin-3-*O*-glucuronide	C_22_H_20_O_13_
unknown saponin. Aglycon: 440.32905 (C_29_H_44_O_3_)	C_58_H_92_O_28_	Chlorogenic acid (3-*O*-Caffeoylquinic acid)	C_16_H_18_O_9_	Dihydroactinidiolide	C_11_H_16_O_2_
unknown saponin. Aglycon: 504.34509 (C_30_H_48_O_6_)	C_41_H_64_O_16_	Chryptochlorogenic acid (4-*O*-Caffeoylquinic acid)	C_16_H_18_O_9_	Dimethoxy-tetrahydroxyflavone	C_17_H_14_O_8_
unknown saponin. Aglycon: 486.33452 (C_29_H_42_O_3_)	C_42_H_64_O_16_	Caffeic acid	C_9_H_8_O_4_	Dihydroxy-methoxyflavone	C_16_H_12_O_5_
unknown saponin. Aglycon: 454.34470 (C_30_H_46_O_3_)	C_47_H_74_O_19_	4-Coumaric acid	C_9_H_8_O_3_	Dimethoxy-trihydroxyflavone isomer 1	C_17_H_14_O_7_
		Sinapic acid	C_11_H_12_O_5_	Trihydroxy-trimethoxyflavone	C_18_H_16_O_8_
		Di-*O*-sinapoylgentiobiose	C_34_H_42_O_19_	Dimethoxy-trihydroxyflavone isomer 2	C_17_H_14_O_7_
		Tri-*O*-sinapoylgentiobiose	C_45_H_52_O_23_	Liquiritigenin (4′,7-Dihydroxyflavanone)	C_15_H_12_O_4_
		Feruloyl-sinapoyldihexoside	C_33_H_40_O_18_	Hymenoxin (5,7,Dihydroxy-3′,4′,6,8-tetramethoxyflavone)	C_19_H_18_O_8_
		Di-*O*-sinapoylglucose	C_28_H_32_O_14_	Epiafzelechin trimethyl ether	C_18_H_20_O_5_
		Feruloyl-disinapoyldihexoside	C_44_H_50_O_22_	Nevadensin (5,7-Dihydroxy-4′,6,8-trimethoxyflavone)	C_18_H_16_O_7_
		Syringaldehyde (3,5-Dimethoxy-4-hydroxybenzaldehyde)	C_9_H_10_O_4_		
		Glucobrassicin (3-Indolylmethyl glucosinolate)	C_16_H_20_N_2_O_9_S_2_		
		3-Methylsulphinylpropyl isothiocyanate	C_5_H_9_NOS_2_		
		4-Methoxy-3-indolylmethyl glucosinolate	C_17_H_22_N_2_O_10_S_2_		
		Sulforaphane	C_6_H_11_NOS_2_		
		Neoglucobrassicin (1-Methoxy-3-indolylmethyl glucosinolate)	C_17_H_22_N_2_O_10_S_2_		
		Scopoletin (7-Hydroxy-6-methoxycoumarin)	C_10_H_8_O_4_		
		***Other phytocompounds***			
		γ-Aminobutyric acid	C_4_H_9_NO_2_		
		Indole-4-carbaldehyde	C_9_H_7_NO		
		Abscisic acid	C_15_H_20_O_4_		
		Kynurenic acid	C_10_H_7_NO_3_		

**Table 5:** Internet search engine (selected) results with keywords “Plant-based foods” and “Plant-based protein”.

Heading of the Source	Address (Internet)/Reference (Patent)	Main Information (Alleged)/Abstract
10 Best Sources of Plant-Based Protein by Whitney E. RD	https://www.house-foods.com/eat-happy/10-best-sources-of-plant-based-protein-by-whitney-e.-rd accessed on 28 November 2022	Protein sources for a plant-based diet (Personal information)
Australian Plant Proteins: Optimized faba bean protein extraction	https://www.csiro.au/en/work-with-us/funding-programs/sme/csiro-kick-start/app accessed on 28 November 2022	Upcoming of plant-based protein products. Disclosure of ongoing research (institutional)
Plant-Based Foods &Proteins Summit Americas	https://bridge2food.com/summits/americas/ accessed on 28 November 2022	Ongoing and upcoming courses and meetings on innovation, business, and industry data on plant-based food and products. Disclosure of information on innovation, market and training
Go Plant-Based with Pistachios	https://americanpistachios.org/nutrition-and-health/the-plant-based-athlete accessed on 28 November 2022 https://americanpistachios.org/sites/default/files/inline-files/GoPlantBasedWithPistachiosFactSheet_112416.pdf accessed on 28 November 2022	Advertisement and information of a organized society of a protein-rich plant source (pistachio)
Plant-Based Protein Market—Global and Canadian Market Analysis	https://nrc.canada.ca/en/research-development/research-collaboration/programs/plant-based-protein-market-global-canadian-market-analysis accessed on 30 November 2022	Executive summary of plant-based protein market, advertisement
Plant-Based Protein Market—Market Insights on Plant-Based Protein covering sales outlook, demand forecast & up-to-date key trends	https://www.futuremarketinsights.com/reports/plant-based-protein-market accessed on 30 November 2022	Plant-based protein market analysis/report, prospective advertisement, and information for financial investment
Plant-based Protein Market Forecast, 2021–2031	https://www.transparencymarketresearch.com/plantbased-protein-market.html accessed on 30 November 2022	Plant-based protein market analysis/report, prospective advertisement, and information for financial investment
Plant-based Protein Market by Type (Soy Protein, Wheat Protein, Pea Protein, Potato Protein, Rice Protein, Corn Protein), Crop Type (GMO), Source Process (Organic), Application (Food and Beverages, Animal Feed, Nutritional Supplements)—Global Forecast to 2028	https://www.meticulousresearch.com/product/plant-based-protein-market-5031 accessed on 30 November 2022	Plant-based protein market analysis/report, prospective advertisement, and information for financial investment
Plant Based Protein Market Worth $23.4 Billion By 2028—Exclusive Report by Meticulous Research^®^	https://www.globenewswire.com/en/news-release/2022/01/03/2360111/0/en/Plant-Based-Protein-Market-Worth-23-4-Billion-By-2028-Exclusive-Report-by-Meticulous-Research.html accessed on 30 November 2022	Plant-based protein market analysis/report, prospective advertisement, and information for financial investment
Plant-based proteins: A growth industry in Canada’s backyard	https://www.edc.ca/en/blog/canada-plant-based-protein-growth.html accessed on 17 January 2023	Plant-based protein market analysis/report, prospective advertisement, and information for financial investment
Plant-based Proteins Market-Market Study on Plant-based Proteins: Popularity of Pea & Wheat Proteins to Rise Faster Than Others	https://www.persistencemarketresearch.com/market-research/plantbased-protein-market.asp accessed on 17 January 2023	Plant-based protein market analysis/report, prospective advertisement, and information for financial investment
Plant-based Foods Market to Hit $162 Billion in Next Decade, Projects Bloomberg Intelligence	https://www.bloomberg.com/company/press/plant-based-foods-market-to-hit-162-billion-in-next-decade-projects-bloomberg-intelligence/ accessed on 28 November 2022	Plant-based protein market analysis/report, prospective advertisement, and information for financial investment
Plant Based Protein Market, By Source (Soybeans, Wheat, Pea, Others), By Type (Isolates, Concentrates, Textured), By Form (Dry Form, Wet Form), By Application, and By Region Forecast to 2030	https://www.emergenresearch.com/industry-report/plant-based-protein-market accessed on 30 November 2022	Plant-based protein market analysis/report, prospective advertisement, and information for financial investment
Alternative Proteins Market	https://www.datamintelligence.com/research-report/alternative-proteins-market accessed on 30 November 2022	Plant-based protein market analysis/report, prospective advertisement, and information for financial investment
Alternative proteins: The race for market share is on	https://www.mckinsey.com/~/media/McKinsey/Industries/Agriculture/Our%20Insights/Alternative%20proteins%20The%20race%20for%20market%20share%20is%20on/Alternative-proteins-The-race-for-market-share-is-on.pdf accessed on 30 November 2022	Plant-based protein market analysis/report, prospective advertisement, and information for financial investment
Plant Based Protein Supplements Market Size, Share & Trends Analysis Report By Raw Material (Soy, Spirulina, Pumpkin Seed, Wheat, Hemp, Rice, Pea, Others), By Product, By Distribution Channel, By Application, By Region, And Segment Forecasts, 2022–2030	https://www.grandviewresearch.com/industry-analysis/plant-based-protein-supplements-market accessed on 17 January 2023	Plant-based protein market analysis/report, prospective advertisement, and information for financial investment
Vegan Protein Market: Global Industry Analysis and Trends (2022–2029) Key Trends, Technology Trends, Market Share and Size	https://www.maximizemarketresearch.com/market-report/global-vegan-protein-market/87218/ accessed on 30 November 2022	Plant-based protein market analysis/report, prospective advertisement, and information for financial investment
Increased Usage of Plant Based Protein for Various Applications is Anticipated to Accelerate the Overall Growth of the Market Further	https://www.databridgemarketresearch.com/press-release/global-plant-based-protein-market accessed on 28 November 2022	Plant-based protein market analysis/report, prospective advertisement, and information for financial investment
Brazil’s Future Farm launches its US expansion with the mission to democratize plant-based meat	https://www.fooddive.com/news/brazils-future-farm-launches-its-us-expansion-with-the-mission-to-democrat/602655/ accessed on 28 November 2022	Plant-based meat advertisement/interview
Latin America & Caribbean: Green finance state of the market 2019 I	https://www.climatebonds.net/resources/reports/latin-america-caribbean-green-finance-state-market-2019 accessed on 26 November 2022	Green bonds market analysis/report, prospective advertisement and information for financial investment
Plant Protein Primer, Exploring the Landscape of Plant Protein Sources for Applications in Plant-Based Meat, Eggs, and Dairy	https://gfi.org/wp-content/uploads/2021/02/2021-02-23_Plant_Protein_Primer_GFI.pdf accessed on 28 November 2022	Presentation, plant species for protein extraction, market analysis
Plant-based Protein Market by Source (Soy, Wheat, and Pea), Type (Isolates, Concentrates, and Textured), Application (Dairy Alternatives, Meat Alternatives, and Performance Nutrition, Animal Feed), and Region (North America, Europe, Asia Pacific, South America, Middle East and Africa), Global trends and forecast from 2019 to 2028	https://exactitudeconsultancy.com/reports/1246/plant-based-protein-market/ accessed on 28 November 2022	Plant-based protein market analysis/report, prospective advertisement, and information for financial investment
The future of plant-based food, according to industry leaders	https://www.veganfoodandliving.com/vegan-business/the-future-of-plant-based-food/ accessed on 30 November 2022	Plant-based food and protein market analysis/report, prospective advertisement, and information for financial investment
Plant-based proteins: building a sustainable future	https://impact.economist.com/perspectives/sites/default/files/plant_based_protein_eiu_infographic.pdf accessed on 30 November 2022	Infographic on Plant-based food and protein market analysis/report, prospective advertisement, and information for financial investment
Plant-Based Innovation For Latin America: Beyond Burgers	https://www.mintel.com/blog/food-market-news/plant-based-innovation-for-latin-america-beyond-burgers accessed on 2 December 2022	
NotCo becomes Chile’s newest unicorn	https://www.leadersleague.com/fr/news/notco-becomes-chile-s-newest-unicorn accessed on 17 January 2023	Financial information of Plant-based food company.
Creating a Sustainable Food Future	https://research.wri.org/wrr-food accessed on 30 November 2022	Folder on sustainable food production from World Resources Institute
You Heard it Here first: The Plant-Based Revolution	https://www.mintel.com/blog/food-market-news/you-heard-it-here-first-predicting-the-plant-based-revolution accessed on 2 December 2022	Data on plant-based food trend
Fazenda do futuro	https://www.fazendafuturo.io/pt-br accessed on 17 January 2023	Home site of Future Farm Company
NotCo	https://notco.com/br/ accessed on 17 January 2023	Home site of NotCo Company
Beyond Meat	https://www.beyondmeat.com/en-US/ accessed on 17 January 2023	Home site of Beyond Meat Company
The New Live Geen Co.	https://thenewbutchers.com.br/nossos-produtos/ accessed on 17 January 2023	Home site of The Live Green Company
	https://www.thelivegreenco.com/ accessed on 17 January 2023	Home site of NotCo Company
**Patents**	**Address (internet)/Reference (patent)**	**Main information (alleged)/Abstract**
Protein Compositions for Plant-Based Food Products and Methods for Making	WO 2021/119498, June, 2021. accessed on 28 November 2022	Disclosed is a method for making protein emulsions for use in making products such as meat substitutes, meat extenders, egg substitutes, dairy analogs, etc., as well as methods for using the emulsion(s) to make various meat substitutes, egg substitutes, dairy analogs etc. Vegetable protein crumbles for use as meat substitutes are also disclosed, either alone or in combination with the emulsion(s).
Protein-Rich micoalgal biomass compositions of optimized sensory quality	US 10,119,947 B2, November, 2018. accessed on 28 November 2022	The invention relates to a method for determining the organoleptic quality of protein-rich microalgal biomass composition, comprising the determination of the content of 11 volatile organic compounds, wherein the 11 volatile organic compounds are pentanal, hexanal, 1-oxten-2one), 3,5-octadien-2-one, nonanal, 2-no-nenal, (E, E)-2,4-nonadienal and hexanoic acid.
